# Supplementary material for: Neurotoxicity in Marine Invertebrates: An Update
Source: Biology (Basel). 2021 Feb 18;10(2):161. doi: 10.3390/biology10020161 (PMC7922589; doi:10.3390/biology10020161)
Supplement: Supplementary file 1 [file biology-10-00161-s001.pdf]

Supplementary Table S1. Effects of pesticides to aquatic marine invertebrates.

Marine organism	Pesticide (class)[1]	Exposure		Toxic/Neurotoxic effects	Ref.
		Dose	Time (h)[2]		
**Echinoderms**					
H. pulcherrimus	MCP (OP)	0.01, 0.1, 1 mg/L	24, 36, 48h	ACh, DA, 5-HT metabolism disruption	Zhang 2017
	MCP (OP)	0.01, 0.1, 1 mg/L	12-48h	HpNetrin/neogenin expression affected	Zhang 2017
	MCP (OP)	5, 10, 30 µg/mL	30, 36, 48, 53h	5-HT-hpr decreased expression, no serotonergic axons, swimming activity affected	Yao 2010
S. purpuratus, S. drobachiensis L. variegatus, S. drobachiensis, S. granularis, D. excentricus	CPS (OP)	1-160 µM	20-30h	Generalized dismorphology	Buznikov 2001; Buznikov 2007
P. lividus	CPS (OP)<br>Basudin (OP)<br>Diazinon (OP)<br>Carbaryl (CA)<br>Pirimicarb (CA)	10^{-4} to 10^{-8}M<br>10^{-3} to 10^{-7}M	24h<br>Up to 30h	Signs of nerve damages<br>Strong AChE activity inhibition	Amaroli 2013<br>Pesando 2003
**Molluscs**					
R. philippinurum<br>*C. gigas*	DDVP (OP)	0.1, 1 mg/L	6h + 42h depuration	Adductor muscles paralysis<br>AChE activity inhibition	Le Bris 1995
C. gigas	DDVP (OP)<br>Carbofuran (CA)<br>Oxamyl (OP)	0.1-200 µM	96h	Reduced AChE activity	Anguiano 2010
	Lindane (OC)	1-2.5 µM	12 days	Slight decreased AChE activity	
	Paraoxon (OP)<br>DFP (OP)<br>Eserine (CA)<br>Carbaryl (CA)<br>Carbofuran (CA)	-	-	Soluble AChE insensitive to OC and CA pesticides	Bocquene 1997
R. decussatus	DDVP (OP)	0.05, 0.25 mg/L	2 days	Oxidative stress<br>Reduced AChE activity	El Ayari 2020
M. edulis	DDVP (OP)	1-10000 µg/L gradient	24h	Increased mortality, reduced AChE activity	McHenery 1997
	AZ (OP)	0.1 mg/L	1h, 24h	Immune system affected, decreased AChE activity	Canty 2007
	Paraoxon (OP)<br>DFP (OP)<br>CPS (OP)<br>Eserine (CA)	0.05-1 mM	24h	AChE activity inhibition	Galloway 2002
	CPS (OP)<br>CPS (OP)	0.003-0.03 mg/L<br>0.4-30 mg/L	24, 48, 96h	Immunotoxic effects, decreased AChE activity	Rickwood and Galloway 2004
Aplysia	Paraoxon (OP)	5 mM	36h	Reduced neurite growth, neural death	Srivatsan 1999
A. californica	Paraoxon (OP)	5 mM	36h	Chlorid conductance inhibition	Filbert 1992
A. juliana	MP (OP)	1, 2 mg/L	7, 14 days	Reduced AChE activity, increased antioxidant enzymes activities	Chen 2014

P. viridis	ATZ (OC)	30-3000 ng/L	7 days	Immune functions alterations, hemolymph AChE activity inhibition	Juhel 2017
**Crustaceans**					
H. gammarus	DDVP (OP)	0.01-100 µg/L<br>0.1-50 µg/L	24h<br>6h	AChE activity inhibition	McHenery 1991<br>McHenery 1997
H. nilssoni	DDVP (OP)	5-320 µg/L	96h	Reduced AChE activity	Murison 1997
L. vannamei	Methamidophos (OP)	0.66-1.35 mg/L	24h	Eye ChE activity inhibition	Garcia-de la Parra 2005
	CPS (OP)	0.7, 1.3 µg/L	4 days	Antioxidant enzymes activities changes; AChE activity inhibition	Duarte-Restrepo 2008
P. serratus	CPS-oxon (OP)<br>Malaoxon (OP)<br>TRZ (OP)<br>DDVP (OP)<br>Paraoxone (OP)<br>Serine sulphate (CA)<br>Carbofuran (CA)<br>Propoxur (CA)<br>Carbaryl (CA)	various	-	Eye ChE enzyme responsiveness	Frasco 2006
	DDVP (OP)	0.057-0.452 µM	24h	Mortality, ChE activity inhibition	Frasco 2008
	Fenitrothion (OP)	39-625 ng/L	96h	Swimming velocity alteration, AChE activity inhibition	Oliveira 2013
	Deltamethrin (PR)	0.6-313 ng/L	96h	Antioxidant enzymes activities changes, Eye AChE activity increase	Oliveira 2012
H. americanus	AZ (OP)	61 ng/L	10 days	Stress/hypoxia changes, oxidative damage, ChE activity inhibition	Couillard 2015
L. salmonis	AZ (OP)	various	-	AChE activity inhibition	Fallang 2004
A. franciscana<br>*A. parthenogenetica*	CPS (OP)	0.1, 1, 5 µg/L	various days	AChE activity inhibition	Varò 2015
A. salina	CPS (OP)<br>Diazinon (OP)<br>Carbaryl (CA)	10^{-11} to 10^{-5}M	72, 96, 192h	Larval survival changes, decreased ChE activity	Gambarella 2018
T. japonicas	CPS (OP)<br>Lindane (OC)	various	24h	AChE activity inhibition	Lee 2105
P. monodon	Deltamethrin (PR)	0.1 µg/L	4 days	Oxidative stress changes, AChE activity reduction	Thi Tu 2012

[1] Pesticides class: OC, organochlorine; OP, organophosphate; CA, carbamate; PR, pyrethroid.
[2]hours of exposure.
5-HT-hpr, serotonin receptor; ACh, acetylcholine; DA, dopamine; 5-HT,serotonin.
ATZ, atrazine; AZ, azamethiphos ; CPS, chlorpyrifos; DDVP, dichlorvos; DFP, diisopropylfluorophosphate; MCP, monocrotophos; MP, methyl parathion; TRZ, triazophos-oxon.

Supplementary Table S2. Effects of microplastics (MPs) to aquatic marine invertebrates.

Marine organism	MP type and size	Exposure: Dose	Exposure: Method and Time (h)[1]	Toxic effects	Neurotoxic effects	Ref.
**Molluscs**						
Mytilus galloprovincialis	PE and PS (100 µm)	1.5 g/L<br>+/- 50 µg/L of PYR	Water<br>7 days	Primary MP accumulation in DG<br>MP presence in G and H<br>DNA damage<br>No oxidative stress<br>Alteration in gene expression (immunity, lysosomal metabolism)	Reduced AChE activity in G, no in H	Avio 2015
	PS (0,11 µm)	0.005, 0.05, 0.5, 5 and 50 mg/L<br>6.3 µg/L Cbz;<br>PS + Cbz (0.05 mg/L + 6.3 g/L)	Water<br>96h	Alteration in gene expression in G (biotrasformation and innate immunity) and in DG (cell stress)<br>DNA damage<br>Oxidative stress in DG	Reduced ChE activity in H	Brandts 2018
Scrobicularia plana	PS (20 µm)	1 mg/L	Water, 21 days (14 days exposure + 7 days of depuration)	Primary MP accumulation in G<br>MP presence also in and DG<br>Oxidative stress in DG<br>Increase of antioxidant capacity in G	Reduced AChE activity in G	Ribeiro 2017
	LDPE (11–13 µm)	1 mg /L<br>+/- 82 ng/g BP-3	Water-sediment, 14 days	Oxidative stress in DG (only LDPE+BP3)<br>Oxidative stress and increase of antioxidant activities in G	No AChE activity inhibition in G	O'Donovan 2020
Donax trunculus	Mixture PP/PE, ratio 1:1 (100-400 mm)	0.06 g/Kg	Sand<br>15 days	Primary MP accumulation in G<br>No MP presence in F<br>Oxidative stress	Reduced AChE activity in G and DG, no in F	Tlili 2020
C. Fluminea	PS-NP fl (80 nm)	0.1, 1 and 5 mg/L	Water<br>96h	Primary MP accumulation in G and in VM<br>Oxidative stress and increase of antioxidant activities<br>Intestinal inflammation	Reduced AChE activity In VM, no in G	Li 2020
Tegillarca granosa	PS-MP (490 ± 11 nm)	1 mg /L<br>+/- 10 and 100 ng/L BPA	Water<br>14 days	Changes in the hematic parameters<br>Immunotoxicity	Increase of neurotrasmitters (GABA, DA, ACh)	Yu Tang 2020

**Crustaceans**						
A. Amphitrite (shrimp)<br>*A.fransiscana (larvae)*	PS (0.1 μm)	0.001, 0.01, 0.1, 1, 10 mg/L	Water<br>24 and 48h	MP accumulation in intestine<br>Swimming alteration<br>Oxidative stress	Partial AChE and PChE activity inhibition	Gambardella 2017
A. fransiscana (shrimp)	PS-NH_2 (50 nm)	0.1-10mg/L	Water<br>48h and 14 days	Alteration in growth and development (48h)<br>Mortality (14 days)<br>Oxidative stress<br>Alteration in gene expression (cell stress, development and molting)	Reduced AChE activity	Varò 2019

[1] hours of exposure.

Invertebrate tissues: DG, digestive gland; F, flesh; G, gills; H, hemolymph; VM, visceral mass.

ACh, acetylcholine; Cbz, carbamazepine; BP-3, oxybenzone; BPA, bisphenol A; DA, dopamine; GABA, g-aminobutyric acid; LDPE, low-density polyethylene; NP fl, fluorescent nanoparticles; PE, Polyethylene; PP, polypropylene; PS, polystyrene; PS-NH_2, amino-modified polystyrene; PYR, pyrene.
